# The First Report of Isolated Clitoral Hood Hair-Thread Tourniquet Syndrome: A Study of Six Patients

**DOI:** 10.7759/cureus.26472

**Published:** 2022-06-30

**Authors:** Ettidal A AlJahdali

**Affiliations:** 1 Obstetrics and Gynecology, King Abdulaziz University Hospital, Jeddah, SAU

**Keywords:** recurrence, pain, pruritus, management, foreskin on clitoris, dysuria, hair-thread tourniquet syndrome

## Abstract

Background and objective

Hair-thread tourniquet syndrome (HTTS) is a rare and potentially dangerous condition that occurs when a hair strand or fabric thread is wrapped around the penis, clitoris, toes, fingers, or other appendages, leading to focal edema, ischemia, and necrosis. This study aimed to examine the cases of six female patients with isolated clitoral hood HTTS.

Methods

This was a retrospective study involving six female patients (age range: two to six years) with isolated clitoral hood tourniquet who presented to the outpatient department (OPD) and emergency room (ER) of the pediatric and adolescent gynecology service at King Abdulaziz University Hospital, Jeddah, Saudi Arabia between January 2010 and December 2021. Data related to patients’ clinical presentation, symptom duration, and management were recorded.

Results

The most common symptom of isolated clitoral hood HTTS in all six cases was pain, followed by redness, itching, discomfort while sitting, and dysuria. Local signs included edema in three cases, tight hair tourniquet around a portion of the clitoral hood in four cases, and loose hair tourniquet around a portion of the clitoral hood in two cases, one of which was during the first episode in a patient who had recurrence (Case 1). Sedation and local anesthetic were used in five cases involving the local removal of four hairs and a strangulated skin. This was followed by local care and antibiotic ointment application. The edges were closed by interrupted stitches in two of the cases as the resulting wounds were broad. Limited clitoral unhooding was performed under general anesthesia in one patient, who had repeated bouts of autoamputation of parts of the hood tissue with resulting disfiguration of the remaining redundant hood, to avoid the additional risk of organ loss. Only two patients experienced recurring episodes.

Conclusions

A high index of suspicion should be maintained when encountering these patients, which can facilitate a prompt resolution to save the affected tissue. HTTS should be suspected in all females presenting with a prominent clitoral hood, genital pain, structural abnormality, swelling, or discomfort. This is the first study to report isolated clitoral hood HTTS; it describes the presentation, manifestation, and management of patients with HTTS. It also addresses preventive strategies to alert mothers and treating physicians of the pediatric and adolescent-age group females to the defect and its underlying causes, particularly when patients have genital structural abnormalities such as excessive clitoral hood labial hypertrophy or ambiguous genitalia.

## Introduction

Hair-thread tourniquet syndrome (HTTS) is a potentially serious condition in which hair or fabric threads get wrapped around an appendage of various body parts (fingers, toes, uvula, tongue, clitoris, labia minor or major, and penis), thereby causing edema, ischemia, and, in severe cases, autoamputation [1]. The first known medical case of HTTS was published in The Lancet in 1832, and the syndrome was termed HTTS in a 1988 pediatric study of six children who presented with the disease [2,3]. Several additional cases of HTTS with varying clinical manifestations have been documented worldwide ever since.

HTTS mostly affects the genitalia, fingers, and toes [3-5]. However, other body-end structures, including nipples, ear lobes, tongue [6], and uvula [7] may also be affected. Genital HTTS is more common in male children, especially circumcised males [8]. There has been an increase in case reports describing clitoral and labial strangulation by hair threads in female children [2,9]. Of note, when the condition is identified and treated early, it is frequently reversible and has no long-term consequences. However, if HTTS is misdiagnosed or not promptly diagnosed, the lack of circulation to the trapped appendage can result in considerable harm to the involved area, including ischemia gangrene, tissue necrosis, and autoamputation [2,5]. Analgesia should be administered when required, and the strangulating hair surgically removed as soon as possible; otherwise, the loss of the strangulated body part is inevitable.

Although the exact cause of genital HTTS is unknown, it has been proposed to be caused by poor hygiene, inadvertent self-infliction, masturbation, or adhesion of wet hair threads to the skin after diaper changing and washing [5,9]. Therefore, child abuse should be considered as a probable cause of genital HTTS. Abuse is a more common cause in pediatric male patients than in females, particularly when caregivers attempt to prevent enuresis [10].

This study aimed to analyze the cases of six females with isolated clitoral HTTS. It is the first study to describe the presentation, manifestation, and management of isolated clitoral hood HTTS. Additionally, the study addressed the preventive strategies to alert mothers and the treating physicians of females of this age group to such a problem and its underlying cause, particularly when the patient has genital structural abnormalities, such as excessive clitoral hood labial hypertrophy or ambiguous genitalia.

## Materials and methods

Study design and setting

This research was approved by the Unit of Biomedical Ethics of King Abdulaziz University in Jeddah, Saudi Arabia. The data of female patients who presented to the outpatient department (OPD) and emergency room (ER) of the pediatric and adolescent gynecology services at King Abdulaziz University Hospital, Jeddah, Saudi Arabia between January 2010 and December 2021 were retrospectively reviewed. These patients had genital HTTS that affected only the clitoral hood without the involvement of the adjacent clitoris or any part of the labia minora.

Inclusion criteria

Female patients who had genital HTTS that had only strangulated the clitoral hood were considered to be included in the study.

Exclusion criteria

Patients who had HTTS involving the clitoris, fingers, toes, or any other body part were not eligible for the study.

Six female pediatric patients who were diagnosed with an isolated clitoral hood hair tourniquet and treated at King Abdulaziz University Hospital were included. Patients' ages ranged from two to six years. The parents of the patients were interviewed to obtain information on the history of child abuse if any, recurrence, and to query if other family members suffered from similar problems. The information provided by the mothers on the symptoms was gathered. The duration of the symptoms ranged anywhere from one to four days. In addition, a thorough careful genital examination was carried out to determine the possible underlining etiology of this unique and uncommon condition; the examination revealed that all patients had prominent clitoral hoods, and one of them had clitoromegaly. The management of these pediatric cases required sedation with local or general anesthesia, followed by variable surgical procedures depending on the presentation.

## Results

**Figure 1:**
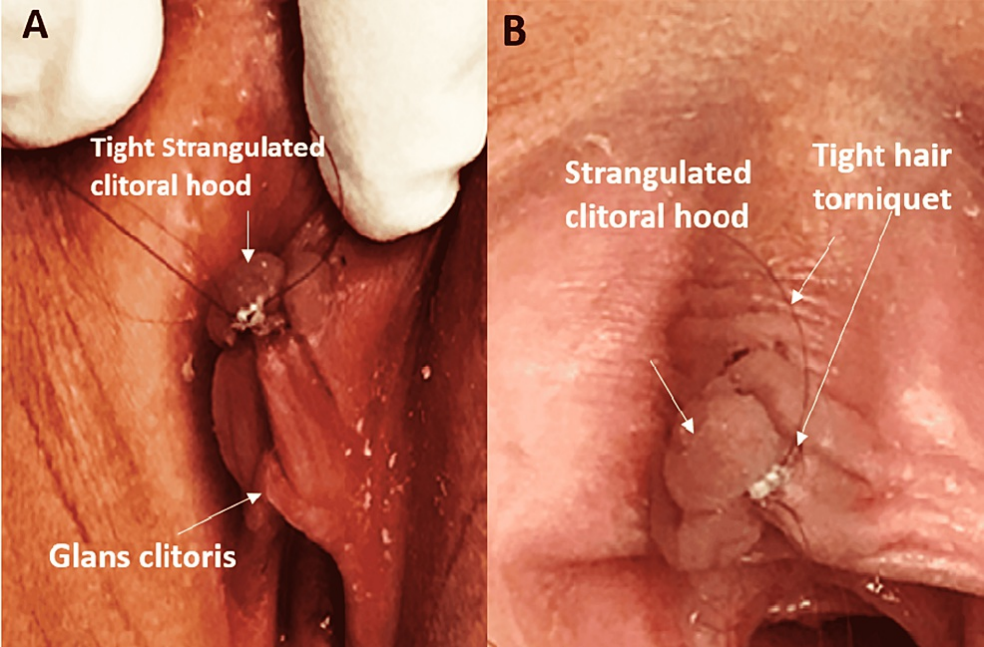
HTTS of clitoral hood: Case 1 The hair was trimmed and the separated skin was removed. The residual stump was treated with simple local care (antibiotic ointment) HTTS: hair-thread tourniquet syndrome

**Figure 2:**
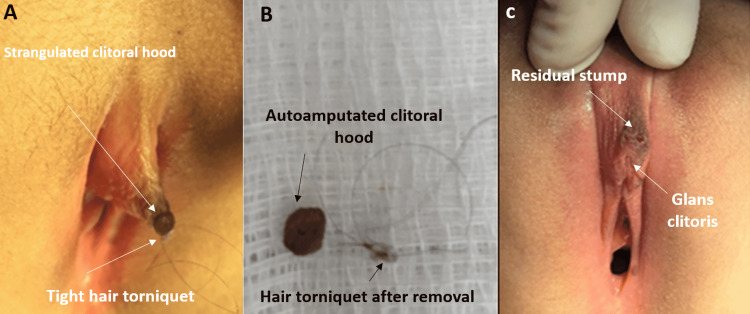
HTTS of clitoral hood: Case 2 The necrotic strangulated part of the hood was easily separated from the hair strand and healed with simple local care (antibiotic ointment) HTTS: hair-thread tourniquet syndrome

**Figure 3:**
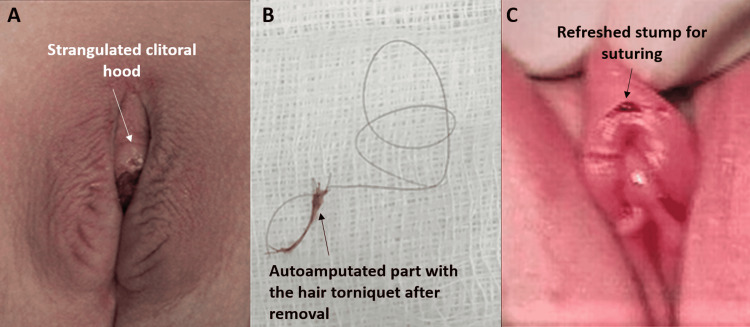
HTTS of clitoral hood: Case 3 Separated necrotic part of the hood with small separation stump. Treated with freshening of the edge, and closed with interrupted stitches and simple local care antibiotic ointment HTTS: hair-thread tourniquet syndrome

**Figure 4:**
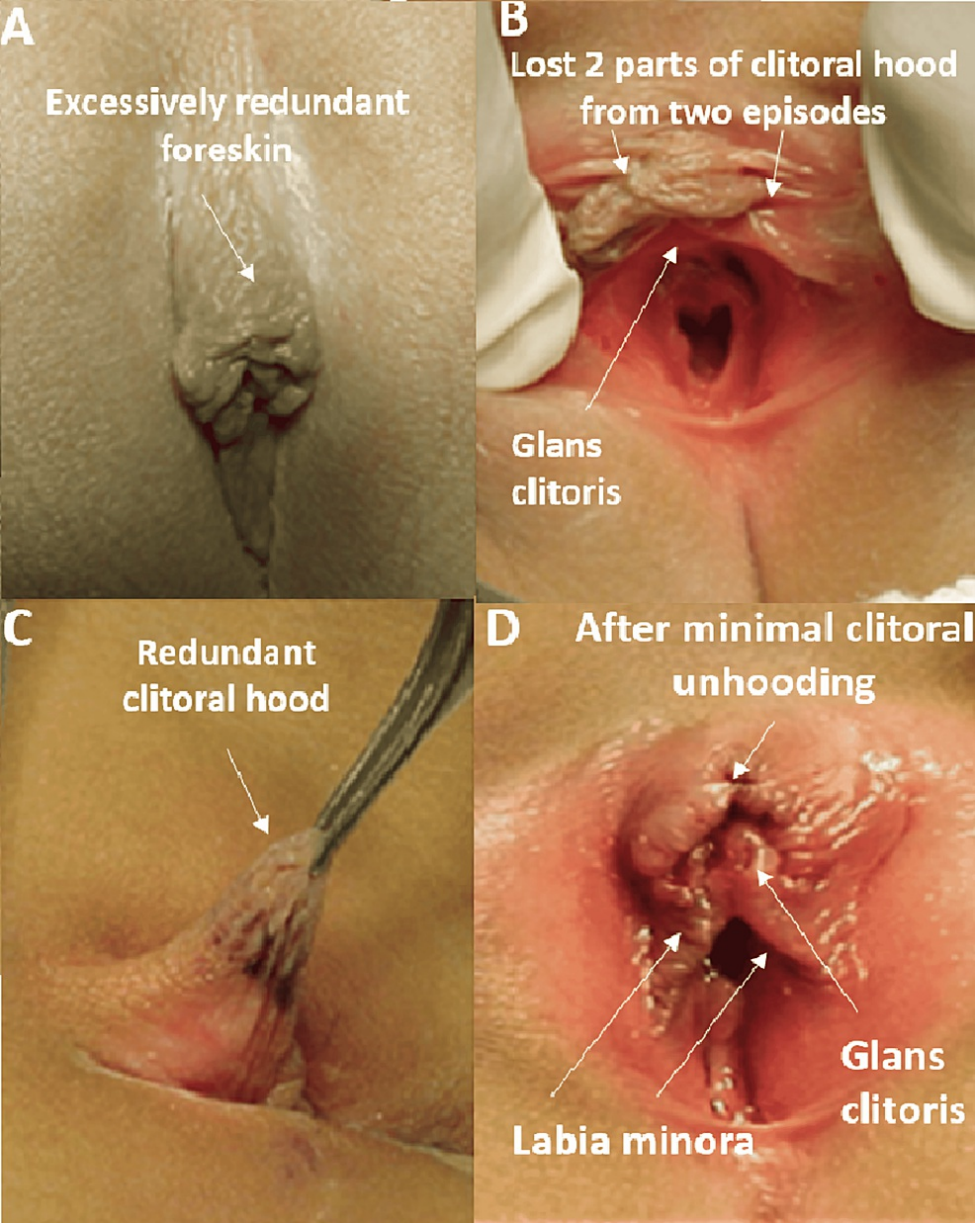
HTTS of clitoral hood: Case 4 History of two episodes of loss of black crust of tissues at the tip of the hood at the time of presentation; examination revealed excessively long scrambled foreskin with a sign of loss of the hood tip (two defects at the tip of the hood). Treated by limited clitoral unhooding of the excess hood HTTS: hair-thread tourniquet syndrome

**Figure 5:**
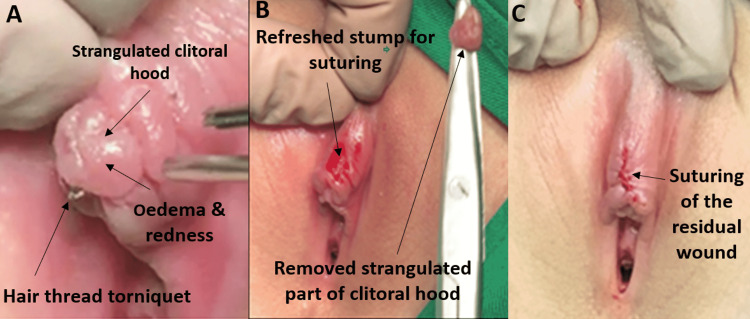
HTTS of clitoral hood: Case 5 Very tight strangulated tip of the hood. Treated by cutting the hair thread base together and suturing the cut edges with interrupted stitches HTTS: hair-thread tourniquet syndrome

**Figure 6:**
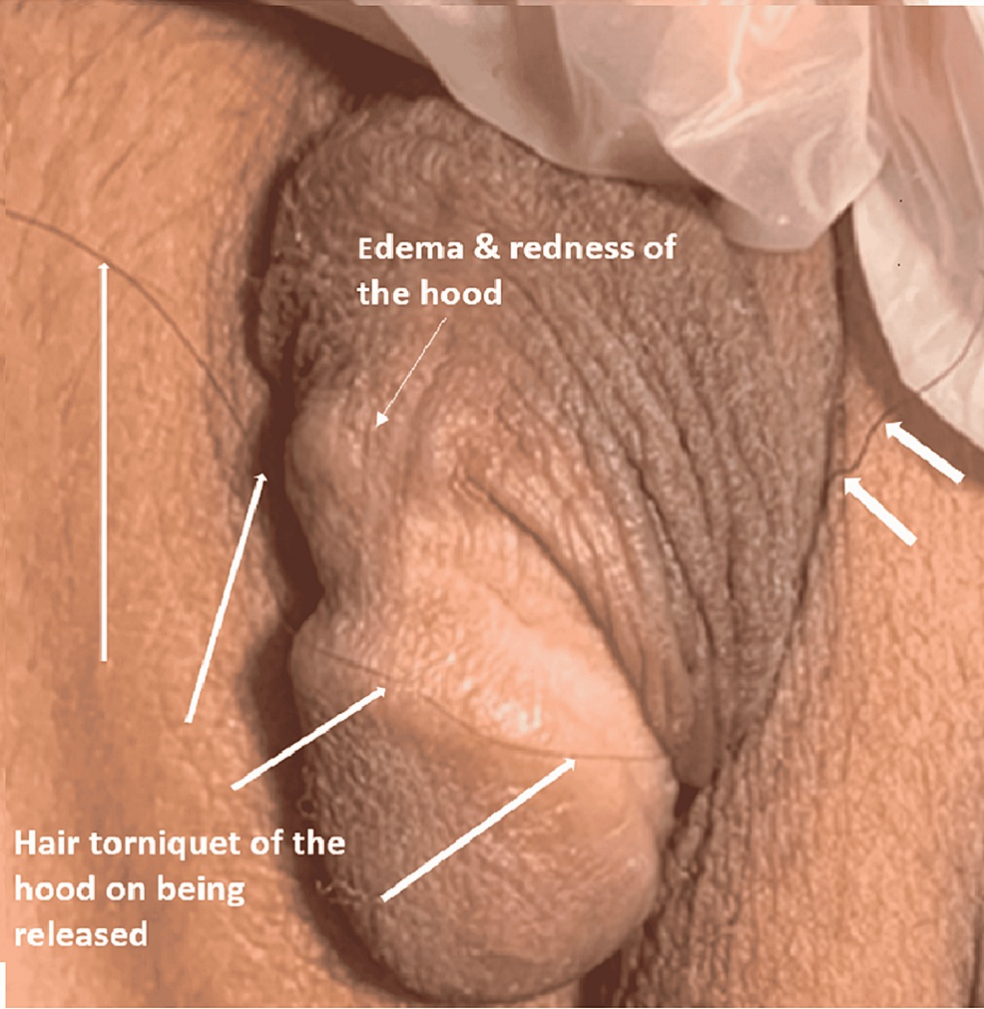
HTTS of clitoral hood: Case 6 Female with ambiguous genitalia seen with a loose strand of hair wrapping the hood causing local irritation with edema and redness. Treated with a simple release of the hair strand, local care, and topical antibiotic HTTS: hair-thread tourniquet syndrome

The most common symptom among the patients in this study was pain. In three of the cases, there was additional redness, itching, discomfort when sitting, and dysuria. The local signs were edema in three cases and a tight hair tourniquet around a part of the clitoral hood in three cases (Figures 1-3); there was a history of another loose wrapping in the first episode of recurrent Case 1, which was released by the mother. Case 4 patient presented late with an irregular tip of disfigured hood due to two episodes of unrealized hood strangulation. The mother gave a history of two episodes with a four-month interval of black tissue mass loss with adherent hair thread in the second episode; unaware of the HTTS possibility, she believed that it was hair that adhered to the infected skin (Figure 4). There was a very tight strangulated tip of the hood that necessitated cutting the hair thread with the strangulated part of the clitoral hood and suturing of the residual stump (Figure 5). In another patient, there was a loose hair tourniquet around a part of the clitoral hood; this patient had ambiguous genitalia (Figure 6).

A local anesthetic cream (Emla) or xylocaine 1% local infiltration with deep conscious sedation was used to remove the hair tourniquet. Sedation and local anesthetic were used in five cases involving the removal of hairs and a strangulated skin. This was then followed by local care and antibiotic ointment application. In two of these cases, the edges were closed by interrupted stitches because the resulting wounds were broad. However, the treatment was conducted under general anesthesia on one patient who had a history of recurrent bouts of autoamputation of necrotic black masses at the tip of the disfigured irregular excessive clitoral hood. This was due to the need to do limited clitoral unhooding of the redundant clitoral hood to avoid further organ loss (Figure 4). Only two patients experienced recurring episodes. A summary of all six cases is provided in Table 1.

**Table 1:** Summary of clinical presentation and treatment of six cases of isolated clitoral hood HTTS HTTS: hair-thread tourniquet syndrome; LA: local anesthesia; GA: general anesthesia

Case number	Age (years)	Number of episodes	Symptoms and signs and disease duration (days)								Management
			Days	Pain	Redness	Itching	Discomfort on sitting	Dysuria	Tightness of hair tourniquet	Edema	
1	6	2	2	Yes	Yes	No	No	No	First time loose, second time tight	Yes	Sedation and LA; hair and strangulated part of skin were removed; local care
2	4	1	2	Yes	No	Yes	Yes	No	Tight	No	Sedation and LA; hair and necrotic tissue were removed; local care
3	2	1	2	Yes	No	Yes	No	Yes	Tight	No	Sedation and LA; hair and autoamputated tissue were removed; the wound was stitched; local care
4	4	2	Not known	Yes	No	Yes	Yes	No	Tight	No	GA; limited clitoral unhooding of the very excessive hood to avoid more serious loss episode of hood and clitoris
5	5	1	1	Yes	Yes	No	No	No	Tight	Yes	Sedation and LA; cutting the base with a hair tourniquet and suturing the cut edges
6	5	1	4	Yes	Yes	No	Yes	Yes	Loose	Yes, ambiguous genitalia	Sedation and LA; release and removal of loose hair; local care

## Discussion

HTTS is a rare and potentially dangerous disorder in which hair or a synthetic fiber part is wrapped around an appendage, resulting in swelling, pain, or even loss of the appendage [11]. HTTS is most commonly reported in children between their first two to six months of life and could be related to maternal postpartum hair loss (telogen effluvium) [12-14], which affects more than 90% of women due to maternal hormonal changes. The exact etiology of hair tourniquet is unclear, and it most commonly occurs accidentally. The potential precipitating factors include autism [10], poor hygiene [5,15], clitoral hypertrophy, anatomic abnormalities, and child abuse, which should be ruled out (this was ruled out in our patients) [16,17]. Clitoral hypertrophy was seen in one of our cases, and an abnormally excessive redundant clitoral hood was apparent in all of our cases. The pathogenesis of hair tourniquet is thought to be due to the high tensile strength of the hair when it is wet, making it more stretchable, thin, and elastic; it becomes entangled around the end structure of the body easily and unnoticeably. As the hair dries, it constricts, resulting in the tourniquet effect [18]. The constriction of hair or thread leads to reduced blood flow and lymphatic drainage, which can cause edema, swelling, and pain, making the hair tighter and more hidden in the edematous entangled part; this makes recognition and diagnosis more difficult [13,14,19]. Delayed diagnosis and intervention may lead to loss of the affected part.

None of the previous studies had reported isolated HTTS of the clitoral hood without the involvement of the glans clitoris, which was the case in all our patients. In this report, all six female patients experienced pain, followed by redness and itching. There was discomfort upon sitting in three cases and dysuria in two cases. The local signs were edema in three cases, tight hair tourniquet around the foreskin on the clitoris in four cases, and loose hair tourniquet around the foreskin on the clitoris in two cases. Our fourth case presented late after two episodes of autoamputation at the tip with a disfigured excessive redundant clitoral hood. One patient had clitoromegaly; however, the hair strand only wrapped the clitoral hood lightly, caused local irritation, and was identified early without causing any damage.

Our findings in these cases demonstrate the importance of proper history taking and careful local examination to evaluate the presence of abnormal congenital or acquired anatomical genital structure and the resulting defects of recurrent episodes. An example of that is the disfigurement observed in all our cases where all of them had abnormal prominent clitoral hood; this necessitated limited clitoral unhooding in Case 4 to correct HTTS predisposing abnormality. Other studies have reported similar experiences in performing labiaplasty in patients with recurrent HTTS of the labia minora with disfigurement of the genitalia that was medically indicated in these cases to avoid further serious organ loss and correction of the deformity. Additionally, in our experience and those of others, the lack of awareness on the part of the mothers about such a serious problem necessitates the need for raising awareness about educational preventive measures to alert mothers to this condition and encourage early reporting of the occurrence of this serious mishap. It also necessitates a high index of suspicion among the treating physicians of this age group of females regarding the defect and its underlying causes, particularly when the patient has genital structural abnormalities such as excessive clitoral hood labial hypertrophy or ambiguous genitalia. The strategic plane of education and prevention of cases of clitoral HTTS is addressed in Table 2. Differential diagnoses should include urinary tract infection, insect bite, dermatitis, colicky infant child abuse, and trauma. Early detection and treatment are critical; however, skin reepithelialization might disguise the hair and lead to a delay in diagnosis.

**Table 2:** Strategic plane of education and prevention of cases of clitoral HTTS HTTS: hair-thread tourniquet syndrome

Age group	The level of care provider
Neonates and infants	General health authority. Written Guidelines for involved professionals in post-natal evaluation with attention to genital HTTS predisposing anatomical abnormality. HTTS prevention conversations at baby health checkups. The baby booklet includes information on educating mothers about HTTS risk related to fallen hair
	Physician awareness of HTTS presentation and differential diagnoses to avoid missing HTTS, which include urinary tract infection, insect bite, dermatitis, colicky infant child abuse, and trauma. Attention to any genital tissue loss or defect that may indicate HTTS
	Nurses, mothers, and caregivers. Protective baby care. Inspect the baby's hands, feet, and genitalia at bath time. Frequent change of socks and mittens. Launder clothes inside out. Separate washing of the clothes of babies. Prompt removal of loose hair from clothing and diaper. Mother's protective care by managing hair loss. Hair should be tied up. Frequent brushing of hair. Dispose of fallen hair
Older children	Educating parents, caregivers, and other involved medical professionals about HTTS' presenting manifestations and predisposing factors to HTTS, and raising awareness about risks, as patients of this age might present differently, and hence evaluation is needed in cases of unexplained swelling and erythema of a finger, toe, or genitourinary manifestation, localized pain and difficulty ambulating in toddlers and small children, and isolated areas of redness and swelling on the fingers, toes, or genital organs without signs of infection. Draw the attention of parents to any HTTS predisposing to anatomical abnormalities, e.g., clitoromegaly, clitoral hood hypertrophy

In 2006, Mat Saad et al. [20] reported 210 cases of HTTS; 44.2% of them involved the penis, 40.4% occurred in the toes, 8.57% in the fingers, and other sites accounted for 6.83% (female external genitalia, uvula, and neck). Among the 210 reported cases, 103 involved the external genitalia, mainly the penis (93 cases), the clitoris (seven cases), labia (two cases), and mons pubis (one case). The median age of the male patients was two years, with two adult males (69- and 77-year-old) who had Alzheimer’s. The median age of the female patients was eight years. Almost all cases (97%) were caused by hair strangulation.

Diaz-Morales et al. reviewed the literature regarding HTTS involving the female genitalia in 2020 [11]. In that review, reports on 33 girls with genital HTTS were obtained, with one patient experiencing HTTS three times. This is similar to two of our patients who had recurrent episodes. The presenting age ranged from six months to 14 years, which is in line with the reported ages of our patients. The most commonly involved parts were labia minora (45.4%), the clitoris (42.4%), labia majora (9.1%), and mons pubis (3%) [21-23].

Adjei et al. (2022) in a systematic literature review [1] reported HTTS cases involving pediatric male and female genitalia spanning almost 70 years. There were 38 female cases from 33 publications (1973-2020) and 147 male cases from 47 publications (1951-2019). The average age of the females was 9.1 ±3.4 years (range: six months to 14 years). In 81% (13/16) of the total cases, the tourniquet was formed by hair threads, with 92% (12/13) of these originating from the patient's body (pubic or scalp hair), and 8% (1/13) from the maternal body. In 19% (3/16) of the total cases, the tourniquet was formed by cotton threads.

The median symptom duration was 2.7 days, with a range of 1-120 hours; an outlier duration of symptoms of two years was reported in one case [24]. Long-term symptoms, including chronic pain, labial defects, and recurrent tourniquets, appeared in cases that involved a duration of symptoms greater than three days. Clitoral hair tourniquets were the most common type, followed by those involving labia minora, labia majora, vagina, and mons pubis.

Table 3 summarizes the details of some of the studies on HTTS including our study of the first six cases of isolated clitoral hood strangulation and other studies of HTTS involving other body parts. The symptoms of HTTS included pain, edema, erythema, abnormal gait, pruritus, discharge, and dysuria [1]. In our experience and those of others [1], HTTS management varied according to the presentation; ranging from simple removal of the strangulating hair with local care in an early presentation to excision of the necrotic lost tissue with the residual stump defect closure if broad in late presentation with necrosis of the strangulated tissue. However, in the presence of predisposing structural abnormalities such as labial hypertrophy or excessively redundant hood, previous reports on labiaplasty of injured or disfigured labia minora due to recurrent HTTS in hypertrophic labia minora [17,25] suggest that the correction of the abnormality should be included in the treatment. A similar approach was taken in Case 4, where limited clitoral unhooding was performed for the patient who had two previous clitoral tissue strangulation confirmed with real defects at the end of a very redundant hood to avoid more serious loss of the clitoris and hood. General anesthesia, local anesthesia, and sedation were used to treat female genital HTTS. The hair-thread tourniquet was excised in 94% (29/31) of the cases, whereas conservative management was used in 6.3% (2/31) of the cases, and spontaneous resolution occurred in one case. Both general and local anesthesias were administered in one case report, in which labiaplasty was also performed [17]. In one case reported from the Caribbean, a packet of white cane sugar was applied to the clitoris, resulting in instant relief of edema and pain and easy removal of the tourniquet with fine forceps [26]. Most cases involving excision of the hair tourniquet reported immediate resolution of symptoms and return to normal anatomy and sensation with no long-term complications. The reported complications include autoamputation of the clitoral hood, hypertrophic clitoris, and labial skin necrosis, with pathological findings revealing benign squamous mucosa with hyperkeratosis, marked acute and chronic inflammation, and focal necrosis [15,16,24].

**Table 3:** Comparison of studies on HTTS HTTS: hair-thread tourniquet syndrome

Authors	Study duration (years)	Patient gender	Study type	Number of cases	Part involved	Age range	Recurrence	Strangulating threads
Mat Saad et al., 2006	1	Males and females	Case report, literature review, and meta-analysis	210	Penis/clitoris/labia/mons pubis/toes/fingers/vulva neck	2–8 years	-	Hair
Bean et al., 2015	10	Males and females	Retrospective study	81	Toes/fingers/genitalia	2 weeks–22 years	-	Hair
Diaz-Morales et al., 2020	14	Females	Case report and literature review	33	Labia minora/clitoris/labia majora/mons pubis	6 months–14 years	3	Hair, cotton
AlJahdali, 2022	12	Females	Retrospective study	6	Isolated clitoral hood	2–6 years	2	Hair
Adjei et al., 2022	70	Males and females	Systematic literature review	185	Clitoris/labia minora/labia majora/vagina/mons pubis	6 months–14 years	-	Hair, cotton

Limitations

One of the limitations of the study is that it involved patients from only a single tertiary hospital. Further multicenter studies are required to gain insights into the magnitude of the problem. Also, this study did not report the differential diagnosis of the presentations and misdiagnosed cases.

## Conclusions

HTTS must be recognized by gynecologists, pediatricians, and family physicians. HTTS is a potentially serious condition that affects critical appendages, such as the genital organs. A high index of suspicion must be maintained to resolve it quickly and prevent delayed diagnosis due to lack of recognition, which could result in significant consequences, such as tissue loss. All girls presenting with a prominent clitoral hood, genital pain, swelling, discomfort, or anatomical anomalies should be suspected of having genital HTTS. The mainstay of treatment for genital HTTS includes rapid identification, removal of the tourniquet, correction of any probable contributing structural defects, and preventive education.
